# Molecularly Imprinted Nanomaterials for Sensor Applications

**DOI:** 10.3390/nano3040615

**Published:** 2013-11-26

**Authors:** Muhammad Irshad, Naseer Iqbal, Adnan Mujahid, Adeel Afzal, Tajamal Hussain, Ahsan Sharif, Ejaz Ahmad, Muhammad Makshoof Athar

**Affiliations:** 1 Institute of Chemistry, University of the Punjab, Quaid-e-Azam Campus, Lahore 54590, Pakistan; E-Mails: leo_star126@yahoo.com (M.I.); tjml786@yahoo.com (T.H.); ahsansharif@pu.edu.pk (A.S.); ejazahmad@pu.edu.pk (E.A.); makshoofathar@pu.edu.pk (M.M.A.); 2 Interdisciplinary Research Centre in Biomedical Materials, COMSATS Institute of Information Technology, Defence Road, Lahore 54000, Pakistan; E-Mails: naseeriqbal@ciitlahore.edu.pk (N.I.); aa@aafzal.com (A.A.); 3 Affiliated Colleges in Hafr Al-Batin, King Fahd University of Petroleum and Minerals, P.O. Box 1803, Hafr Al-Batin 31991, Saudi Arabia; E-Mail: adeelafzal@kfupm.edu.sa

**Keywords:** molecularly imprinted polymers, nanotechnology, sensors, nanomaterials

## Abstract

Molecular imprinting is a well-established technology to mimic antibody-antigen interaction in a synthetic platform. Molecularly imprinted polymers and nanomaterials usually possess outstanding recognition capabilities. Imprinted nanostructured materials are characterized by their small sizes, large reactive surface area and, most importantly, with rapid and specific analysis of analytes due to the formation of template driven recognition cavities within the matrix. The excellent recognition and selectivity offered by this class of materials towards a target analyte have found applications in many areas, such as separation science, analysis of organic pollutants in water, environmental analysis of trace gases, chemical or biological sensors, biochemical assays, fabricating artificial receptors, nanotechnology, *etc.* We present here a concise overview and recent developments in nanostructured imprinted materials with respect to various sensor systems, e.g., electrochemical, optical and mass sensitive, *etc.* Finally, in light of recent studies, we conclude the article with future perspectives and foreseen applications of imprinted nanomaterials in chemical sensors.

## 1. Introduction

**Figure 1:**
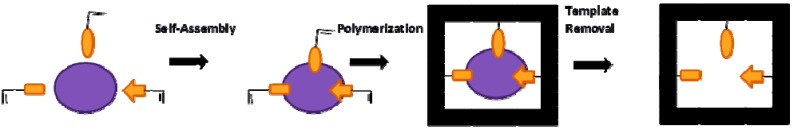
Schematic diagram of molecular imprinting.

The molecular imprinting technique produces complex polymers with template-specific interaction sites or cavities within the polymer structure. These specific recognition cavities can be fabricated for neutral to charge-bearing analytes/templates. The imprinting process involves the organization of polymerizable monomer molecules around a template or imprint molecule, followed by the polymerization in the presence of a cross-linker. The monomer should contain complimentary functionalities with respect to the template molecule. The extraction of the template after polymerization results in the formation of imprinted cavities inside the 3D-matrix of the polymer. The resulting polymer is known as imprinted polymer, which selectively binds the template molecule, when subjected to a solution of the template molecule or a mixture of the template and analogous molecules [1]. A schematic representation of molecular imprinting is shown in Figure 1. Imprinting is a very useful method to serve the purpose of molecular recognition. When combined with nanotechnology, molecularly imprinted nanomaterials exhibit enhanced sensitivity and selectivity, due to the added advantages of both techniques, which may eventually lead to the development of more suitable matrices for desired applications [2].

A large number of imprinted nanomaterials have already been developed, such as imprinted nanoparticles (Imp-NPs) [3], imprinted nanocomposites (Imp-NCs) [4] and imprinted hybrid materials [5], for a variety of applications, such as separation science [6], molecular recognition [7] and chemical sensing of different analytes [8]. Imprinted nanomaterials have gained much importance, due to their foremost advantages, such as high surface area, large number of predetermined recognition sites, high chemical and thermal stability, comparative simplicity, low preparation cost and potential application to a variety of analytes [9,10]. The ease of preparing imprinted nanomaterials and straightforward compliance also play a decisive role in achieving greater success as compared to other receptors [11]. Molecular imprinting has a wide range of potential applications, for instance, in clinical diagnostics [12,13], selective adsorption and separation [14], drug delivery [15], solid-phase extraction [16], synthesis and catalysis [17], environmental analysis [18], enantioselective recognition [19] and, most importantly, chemical sensing [20]. Thereby, imprinted materials with nanoscale dimensions have significantly contributed to these fields and can potentially have a greater impact on nanoscience and technology in the future.

A wide range of imprinted nanomaterial morphologies, including nanospheres, nanocapsules, nanoshells, nanoclusters, nanocrystals, nanorods, nanofibers and nanoparticles, *etc.*, have been developed for different paradigms [21,22,23]. The shape and size of the ultimately imprinted nanomaterial principally depends on the synthetic conditions and methodology. However, the nature of bonding interactions [24] and the compatibility between the template and monomers in a typical polymer matrix play an important role. A number of different methods are reported in the literature for the generation of imprinted nanomaterials, including bulk, suspension, multi-step swelling, mini-emulsion, core-shell and precipitation polymerizations [25].

The immobilization of a sensitive material on the transducer interface is also important in the development of a typical sensor device, which has been realized through spin or spray coating, sputtering, Langmuir–Blodgett and other methods [5,16,18,19,20,25]. The selection of a transducer device, on the other hand, mainly depends on the nature of interactions between the analyte of interest and the sensing material, because the characteristic changes in physical or chemical properties of sensing material are determined upon its exposure to the analyte itself or a mixture of analyte and analogous molecules. Rapid response, high sensitivity, signal stability, miniaturized size and robustness to external parameters are salient features of a model transducer device, while its adaptability for on-field measurements is also valuable for real-time applications. Different types of transducers have been combined with imprinted nanomaterials for developing chemical sensors. Nonetheless, the primary goal of a transducer is to monitor binding events for providing reliable signal output. In this article, the type and nature of different imprinted materials, a comparison of their morphology and ultimate applications in different fields are exclusively discussed along with their potential growth, application and future prospects.

## 2. Molecular Imprinted Nanomaterials

### 2.1. Imprinted Nanoparticles (Imp-NPs)

Imp-NPs are suitable candidates for fabricating sensing layers. They possess a high surface area and a small size. The collaboration of nanotechnology with molecular imprinting provides a better alternative for the monitoring and detection of different analytes, due to their high sensitivity and selectivity at a trace level. Molecularly Imp-NPs have been synthesized by various researchers using different techniques. However, synthesis of Imp-NPs is not as straightforward as anticipated, since many factors control their physicochemical properties, such as the choice of monomer, the degree of cross-linking, the extent of interaction with the template, the removal of the analyte, *etc.* Familiar techniques for the synthesis of Imp-NPs are precipitation, mini- and micro-emulsion, core-shell and free radical polymerization [26]. Herein, we discuss different strategies from the literature.

Precipitation polymerization is a commonly adopted method for the synthesis of Imp-NPs. Yoshimatsu *et al.* [27] explained the precise control over the size of NPs. They prepared NPs of different sizes in the range of 100 nm–2.5 μm by varying concentration of two cross-linking monomers. This was the foremost advantage of their strategy, which ultimately helped in chiral recognition of racemic propranolol templates, *i.e.*, Imp-NPs showed 20-folds higher affinity for (*S*)-propranolol compared to (*R*)-propranolol. Precipitation polymerization was also employed to develop NPs of smaller dimensions in a report published by Xiao *et al.* [28]. The authors used constituents, *i.e.*, methacrylic acid as the functional monomer, 2-ethyl-2-(hydroxymethyl) propane-l,3-diol trimethacrylate as the cross-linker and selected fluoroquinolones (levofloxacin, ofloxacin and ciprofloxacin) as the templates, respectively. They fabricated Imp-NPs with dimensions of 50–100 nm. Analytical measurements revealed selective recital of Imp-NPs for levofloxacin and its analogues in comparison with non-imprinted ones. The binding capacity of Imp-NPs was higher for the target analyte, *i.e.*, levofloxacin, as compared to its structural analogues [28], either observed in acetonitrile or water.

In another approach, Liu *et al.* [29] used precipitation polymerization to yield even smaller Imp-NPs, *i.e.*, 50–80 nm, for enantiomeric identification of *d-zopiclone*. Imp-NPs were produced by dilution of pre-polymerization mixtures, while optimizing a number of parameters, such as the template to monomer ratio, the type and amount of the cross-linking monomer, the functional monomer to cross-linker ratios, the pH and the concentration of salts for buffers [29]. Other interesting strategies for diverse applications are also reported. For instance, ion imprinted nanomaterials, *i.e.*, Imp-NPs of sizes 60–100 nm for the detection of Cu(II) ions [30] and Imp-NPs of dimensions 50–90 nm for the analysis of Cs(I) ions [31] were synthesized using a similar methodology. Experimental studies revealed that Imp-NPs exhibited pronounced selectivity and rapid response towards respective target species. Therefore, these results are comprehensive with respect to the aforementioned traits of imprinted nanomaterials and their application in chemical analysis.

Emulsion/suspension polymerization has also been used for the preparation of Imp-NPs. One such example is reported by Gu *et al.* [32], who fabricated chlorogenic acid (CGA) surface-imprinted magnetic polymer nanoparticles through water-in-oil-in-water multiple emulsion suspension polymerization. The core-shell configuration of 50 nm Imp-NPs and their reusability and regeneration with good rebinding capability, *i.e.*, up to 78.85% upon the fifth exploitation, were the salient features of these Imp-NPs. It was revealed that magnetic susceptibility was achieved by successful encapsulation of Fe_3_O_4_ NPs with a high encapsulation efficiency, *i.e.*, 19.3 wt%. Furthermore, Imp-NPs demonstrated appreciable sensitivity and selectivity towards CGA in contrast to the interfering analyte, caffeic acid (CFA). Imp-NPs showed three-folds better response as compared to non-imprinted NPs, whilst the CGA to CFA recognition ability of Imp-NPs was 6.06-times higher as compared to non-imprinted nanostructures [32].

**Figure 2:**
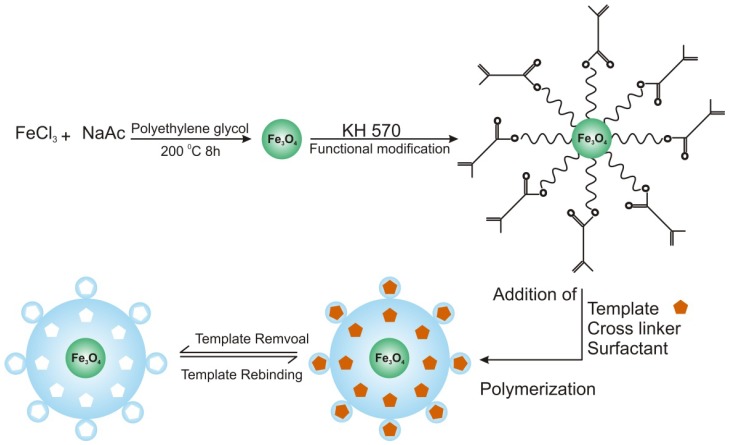
Schematic representation of synthetic route for magnetic imprinted nanoparticles (Imp-NPs). Adopted from [33].

In another study, Dai *et al.* [33] presented the fabrication of magnetic Imp-NPs through the atom transfer radical emulsion polymerization (ATREP) technique. The magnetic Imp-NPs were produced for tetracycline (TC) detection in aqueous medium. Magnetic Imp-NPs demonstrated suitable thermal stability and super paramagnetic behavior for fast recognition of the template. Figure 2 illustrates the schematic for the synthesis of magnetic Imp-NPs: firstly, Fe_2_O_3_ NPs are obtained from FeCl_3_ by thermal decomposition, and subsequently, KH570 (silane coupling agent) vinyl monomers are immobilized on the nanoparticles’ surface and are polymerized to restrict the leakage of Fe_2_O_3_. Finally, ATREP in the presence of water and surfactant yields nanosized imprinted particles [33]. The analytical measurements showed selective recognition of TC over the competitive antibiotics through template-specific affinity interactions. In addition, rebinding experiments revealed that the Imp-NPs possessed specific adsorption equilibrium, kinetics and selective template recognition capabilities. From the Langmuir isotherm, the estimated adsorption capacity of Imp-NPs towards TC was found to be 12.10 mg g^−1^ at 298 K, which was claimed to be 6.33-times higher than magnetic non-imprinted nanoparticles.

The mini-emulsion polymerization technique also produces selective binding sites for Imp-NPs for carbamazepine with higher binding selectivity for the analyte [34]. In a recent study [35], a comparison of micro-emulsion and suspension polymerization was performed, and it was found that Imp-NPs obtained through the latter approach had higher affinity towards the template, when fabricated on potentiometric electrodes, as exhibited by a wide-range linear response and greater selectivity.

Another strategy for the synthesis of soluble Imp-NPs is iniferter polymerization, which offers selectivity similar to that of natural antibodies. Imp-NPs emerged as a suitable substitute to natural receptors by demonstrating a longer shelf life and higher affinity for the template [36,37]. The core-shell approach has been explored by many groups for the synthesis of Imp-NPs. In one such study, imprinted cavities were formed in an ethylene glycol dimethacrylate (EDGMA) shell for the recognition of cholesterol. The core-shell particles had an average size of 76 nm. Template imprinting in super-paramagnetic polymer and magnetite ferrocolloid cores was also investigated, which exhibited similar efficiency [38]. Novel Imp-NPs on a Fe_3_O_4_ magnetic core were also synthesized, which offered excellent binding capacity for chrysoidine. These core-shell Imp-NPs were subsequently used for developing chemiluminescence sensors for the analysis of food samples [39].

Highly selective core-shell Imp-NPs for β-estradiol on silica were synthesized by Ma *et al.* [40], which exhibited fast kinetics and high selectivity towards the template. The authors proposed that these core-shell Imp-NPs could be used for developing an imprinted membrane sensor. Bovine hemoglobin protein Imp-NPs with a magnetic polystyrene core were fabricated using multistep core-shell polymerization mechanism with 3-aminophenylboronic acid as the monomer. Imp-NPs were more robust and reusable, because the selected monomer was grafted tightly on polystyrene via aromatic ring electron-pairing interactions [41]. Other techniques used for the fabrication of Imp-NPs include the sol-gel process [42], bulk polymerization [43], covalent grafting [44] and electropolymerization [45] for achieving high selectivity and sensor response.

### 2.2. Imprinted Nanospheres

Precipitation polymerization is the most widely used method for the synthesis of imprinted nanospheres for the recognition of desired species [46]. Precise control over the synthesis of Imp-nanospheres is achieved via precipitation polymerization with varying ratios of two different cross-linkers. The final size of the Imp-spheres varies from 100 nm to 2.5 μm, and the resultant imprinted sites exhibited a high chiral selectivity [27]. Imp-nanospheres having a size of roughly 450 nm were synthesized for the selective recognition of di(2-ethylhexyl)phthalate by precipitation polymerization. The resultant nanospheres showed substantial binding capacity with the analyte and, therefore, were used as the recognition layer in sensor applications [47]. A one-step procedure for the synthesis of Imp-nanospheres via precipitation polymerization was developed for 17β-estradiol, which yielded Imp-nanospheres with regular spherical morphology. HPLC analyses revealed that the produced nanospheres had better selectivity than the nanospheres produced through the conventional polymerization technique [48]. Titania hybrid Imp-nanospheres were fabricated by precipitation reaction using 3-(trimethoxysilyl)propyl methacrylate as the coupling agent. The results indicated their faster adsorption kinetics and that they were an attractive choice for sensor application [49]. Mini-emulsion polymerization is another promising alternative for the synthesis of Imp-nanospheres, which leads to the development of affinity-based [50] optical sensors [51].

### 2.3. Imprinted Nanoshells

Molecularly imprinted nanomaterials as a recognition element for sensors have been progressively applied, e.g., for selective recognition of guanosine and its analogous compounds. The polymer matrix was synthesized using methacryloylamido-cysteine attached to a CdS quantum dots reconstructed surface shell through the synthetic host polymerization method [52]. In another study, novel Au-Ag nanoshells were prepared for binding dipicolinic acid and its analogue, *i.e.*, *Bacillus cereus*. Imp-nanoshells were fabricated by reconstruction of a methacryloylamido-cysteine monolayer attached with Au-Ag nanoclusters. The resultant nanoshells showed high affinity towards the target analyte, which was recorded by a sensor response in terms of decreasing fluorescence intensity [53]. In a similar approach, Imp-nanoshells for binding dipicolinic acid and its analogue, *Bacillus cereus*, were fabricated with polymerizable methacryloylamido-cysteine attached to gold nanoparticles (Au-NPs), which demonstrated a good sensor response as a function of decreasing fluorescence intensity [54].

### 2.4. MIP Nanofibers

Nanofibers are nanostructures with diameters in the nanometer range. They have been resourcefully used in sensing applications. Piperno *et al.* [55] immobilized polymer nanofibers in Imp-NPs for an amino acid derivative and used them in a fluorescence-based sensor for dansyl-L-phenylalanine detection. Poly(vinyl alcohol) was used as the supporting material with Imp-NPs, because it was water-soluble and could be spun into very thin fibers. The resulting nanofibers showed first-rate bind efficacy with the target molecule and good selectivity as demonstrated through competitive binding experiments with the non-fluorescent analogues, boc-L-phenylalanine and boc-D-phenylalanine [55]. In a different approach [56], polyimide nanofibers were fabricated with estrone Imp-NPs via the electrospinning technique. This strategy showed excellent specific recognition and adsorption kinetics for estrone. The success behind this approach was in retaining the high aspect ratio and surface area provided by the binding sites . Nanofibers have also been used specifically for chiral recognition, *i.e.*, identification and discrimination among enantiomers, for instance; nanofibers have been used in membrane synthesis [57,58]. An Imp-nanofiber membrane was synthesized using N-α-benzyloxycarbonyl-D-glutamic acid (Z-D-Glu) or N-α-benzyloxycarbonyl-L-glutamic acid (Z-L-Glu) as imprint molecules. Enantiomers selectively adsorbed on the respective nanofiber membrane without depression of permselectivity, which provided an effective means of separating enantiomers with high throughput [57]. Cellulose acetate was also used to synthesize Imp-nanofiber membranes by the electrospray method for the same analytes discussed above. The performance of the imprinted membrane and the nanofiber imprinted membrane was evaluated in view of the affinity constant, the adsorption selectivity and the permselectivity. It was found that Imp-nanofibers had augmented permselectivity and flux [58].

A brief description and comparative analysis of imprinted nanomaterials is also presented in Table 1 for a quick overview.

**Table 1:** Selected examples of different types of molecularly imprinted nanomaterials synthesized by various approaches, their particle size and for intended targets are summarized here.

Nanomaterials	Synthesis	Size	Analytes	Ref.
**Nanoparticles**	Precipitation	100 nm–2.5 μm	(*S*)-propranolol	[27]
		50–100 nm	Levofloxacin and fluoroquinolone	[28]
		50–80 nm	d-zopiclone	[29]
		60–100 nm	Cu(II)	[30]
		50–90 nm	Cs(I)	[31]
	Emulsion-suspension	50 nm	Chlorogenic acid	[32]
	Emulsion	50–200 nm	Tetracycline	[33]
	Miniemulsion	180–251 nm	Carbamazepine	[34]
	Microemulsion	40–100 nm	Promethazine	[35]
	Iniferter polymerization	25–106 μm	Acetoguanamine	[36]
	Core-shell emulsion	76 nm	Cholesterol	[38]
	Core-shell	–	Chrysoidine	[39]
	Core-shell	–	17β-estradiol	[40]
	Core-shell	480 nm	Hemoglobin	[41]
	Precipitation	450 nm	di(2-ethylhexyl) phthalate	[47]
	Precipitation	3μm–400 nm	17β-estradiol	[48]
	Precipitation	100–200 nm	Bensulfuron-methyl	[49]
	Miniemulsion	240–255 nm	L-Boc-phenylalanine anilide and L-Boc-phenylalanine	[50]
**Nanoshells**	Thiol ligand capping method	45 nm	Guanosine	[52]
	Thiol ligand capping method	16 nm	Dipicolinic acid	[53]
	Thiol ligand capping method	13 nm	Dipicolinic acid	[54]
**Nanofibers**	Precipitation	400 nm	dansyl-L-phenylalanine	[55]
	Electrospinning	150 nm	Estrone	[56]
	Electrospray deposition	165–564 nm	N-α-benzyloxycarbonyl-D-glutamic acid and N-α-benzyloxycarbonyl-L-glutamic acid	[57]
	Electrospray deposition	200–500 nm	N-α-benzyloxycarbonyl-D-glutamic acid and N-α-benzyloxycarbonyl-L-glutamic acid	[58]

## 3. Selected Sensor Applications of Imprinted Nanomaterials

Herein, we present numerous applications of the aforementioned molecularly imprinted nanomaterials in different sensors. Table 2 also provides detailed information about these devices with respect to the type of sensor and the analytes tested and the limits of detection achieved.

**Table 2:** Selected examples of various sensors using molecularly imprinted nanomaterials: explaining the underlying principle, the nature of the sensing material, the target analytes and the detection ranges. QCM, quartz crystal microbalance; MWCNT, multi-walled carbon nanotube.

Sensors	Transducer Type	Nanomaterial	Target Analytes	Detection Range	Ref.
**Electrochemical**	Square wave voltammetry	Nanoparticles	Promethazine	1.0 × 10^−8^–1.0 × 10^−2^ M	[35]
	Cyclic volammetry	Au- nanoparticles	Theophylline	4 × 10^−7^–3.4 × 10^−3^ mol L^−1^	[59]
	Cyclic volammetry	Au- nanoparticles	Bisphenol A	8.0 × 10^−6^–6.0 × 10^−2^ mol L^−1^	[60]
	Cyclic volammetry	Au- nanoparticles/MWCNTs	Bisphenol A	1.13 × 10^−7^–8.21 × 10^−3^ mol L^−1^	[61]
	Cyclic volammetry	Au- nanoparticles	Trinitrotoluene	46 ppt	[62]
	Cyclic volammetry	Au-nanoparticles	Dopamine	10^−6^–10^−9^ M	[63]
	Electrochemical	Molecularly imprinted polymer/MWCNTs	Dopamine	5.0 × 10^−7^–2.0 × 10^−4^ mol L^−1^	[64]
	Cyclic voltammetry/amperometry	Au- nanoparticles/MWCNTs	Chlortetracycline	9.0 × 10^−8^–5.0 × 10^−5^ mol L^−1^	[65]
	Cyclic voltammetry/differential pulse voltammetry	PbS/Au coated Fe_3_O_4_ nanoparticles/MWCNTs	Streptomycin	1.0 × 10^−6^–1.0 × 10^−3^ mol L^−1^	[66]
	Cyclic voltammetry	Ag-nanoparticles	Dimethoate	1.0-1000 ng mL^−1^ and 1.0-50 μg mL^−1^	[67]
	Cyclic voltammetry	Core-shell nanoparticles	Tert-Butylhydroquinone	0.1–50.0 mg kg^−1^	[68]
	Cyclic voltammetry	Chitosan-Pt /graphene-gold nanoparticles	Erythromycin	7.0 × 10^−8^–9.0 × 10^−5^ mol L^−1^	[69]
	Electrochemiluminescence	Nanoparticles	Thifensulfuron-methyl	5.0 × 10^−10^–1.0 × 10^−7^ M	[70]
**Optical**	Chemiluminescence	Nanoparticles	Chrysoidine	1.0 × 10^−4^–2.0 × 10^−6^ mol L^−1^	[39]
	Reflectometric interference spectroscopy (RIfS)	Nanospheres	L-Boc-phenylalanine anilide	0.4–1.65 mM	[51]
	Fluorescence	Nanocrystals	Guanosine	50–800 µg L^−1^	[52]
	Fluorescence	Au/Ag- nanoclusters	Dipicolinic acid	–	[53]
	Fluorescence	Au-nanoparticles	Dipicolinic acid	10^−7^–10^−4^ mol L^−1^	[54]
	Fluorescence	Nanofibers	Dansyl-L-phenylalanine	10 μM–1 mM	[55]
	Fluorescence	Au/Ag nanoclusters	Dipicolinic acid	–	[71]
	Fluorescence	Quantum dot/imprinted polymer composite	Salivary proteins	0.4–2.68 mg mL^−1^	[72]
	Chemiluminescence	Fe_3_O_4_ nanoparticles	Lysozyme	5–2000 ng mL^−1^	[73]
	Fluorescence	Core shell nanoparticles	Naphthalene	–	[74]
**Mass-sensitive**	QCM	Nanoparticles	17β-estradiol	3.67 nM–3.67 pM	[75]
	QCM	Nanoparticles	Folic acid	1–30 ppm	[76]
	QCM	Nanoparticles	Lysozyme	0.2–1500 μg mL^−1^ and 460–1500 ng mL^−1^	[77]
	QCM	Nanoparticles	Peptide	90–900 nM	[78]

### 3.1. Electrochemical Sensors

#### 3.1.1. Bioanalysis

**Figure 3:**
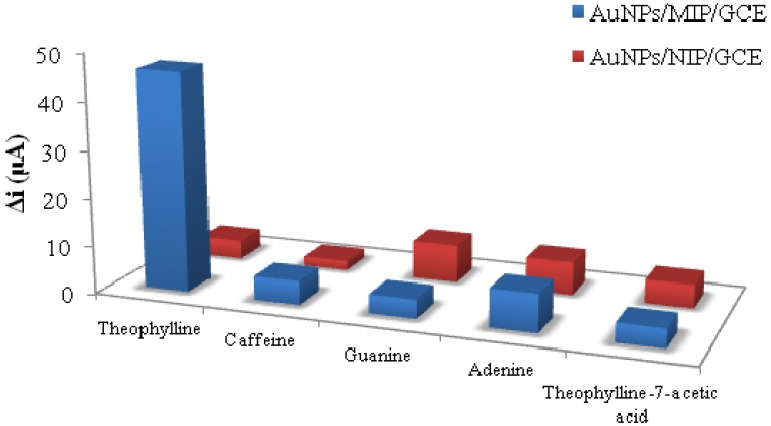
Selectivity comparison of Au-NPs/MIP/GCE (glassy carbon electrode), adopted from [59]. Use parenthesis for µA.

For the first time, Mosbach and coworkers [79] introduced the concept of electrochemical sensors based on imprinted materials. They designed a phenylalanine anilide sensor that worked as a field effect capacitor, *i.e.*, a reduction in capacitance was observed upon exposure to the target analyte. Later, an electrochemical sensor using gold nanoparticles (Au-NPs) was extensively fabricated by many researchers for chemical analysis [59,60,61,62]. Au-NPs were deposited on a pre-formed layer of an electro-polymerized o-phenylenediamine coated glassy carbon electrode. The experimental studies revealed that the sensor exhibited high sensitivity and selectivity with a suitable detection limit of 1.0 × 10^−7^ mol L^−1^ for theophylline. The selectivity studies are revealed in Figure 3, where the response of interfering species along with the target analyte is shown [59].

**Figure 4:**
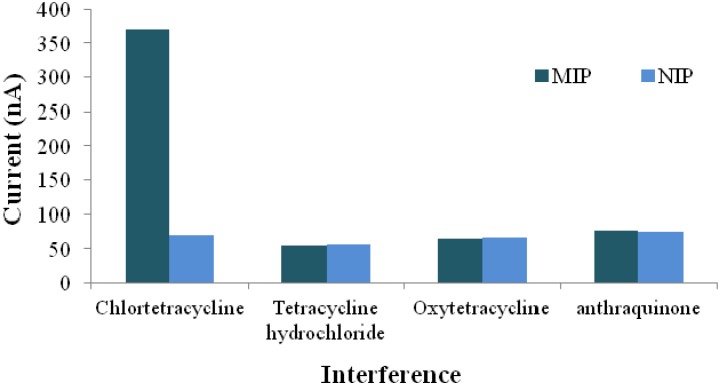
Current responses of the chlortetracycline-imprinted and non-imprinted polymer (NIP) for different analytes, adopted from [65].

Gam-Derouich *et al*. [63] fabricated a highly sensitive electrochemical sensor for dopamine by immobilizing Au-NPs on a gold electrode using mercaptobenzene diazonium salt as a coupling agent. MIP film developed by photo-polymerization resulted in a highly specific and selective recognition of dopamine with a detection limit down to 0.35 nmol L^−1^. This enhanced sensitivity was attributed to the large surface area along with the catalytic effects of Imp-NPs [63]. Dopamine detection was also reported by Kan *et al*. [64]: a sensor for dopamine was made by using a composite of MWCNTs with dopamine imprinted polymer on a glassy carbon electrode. The sensor achieved equilibrium in 30 min and showed remarkable sensitivity and selectivity, with a linear response range of 5.0 × 10^−7^ to 2.0 × 10^−4^ M [64]. An electrochemical sensor based on a composite of β-cyclodextrin-MWCNTs and Au-NPs polyamide amine dendrimer nanocomposites was developed for selective determination of chlortetracycline (CTC). The sensor was fabricated by modifying a gold electrode with the above-mentioned composites with high selectivity and stability towards the target analyte. The sensor exhibited a detection range from 9.0 × 10^−8^ to 5.0 × 10^−5^ mol L^−1^ and a detection limit of 4.954 × 10^−8^ mol L^−1^. The selectivity trend for CTC in the presence of interfering species of the same concentration is shown in the Figure 4 [65].

A highly selective potentiometric sensor for promethazine was fabricated by two different polymerization techniques, *i.e.*, microemulsion and suspension polymerization. A sensor with a suspension polymerized layer showed more affinity for the template with a Nernstian response (31.25 ± 0.8 mV decade^−1^) in the range of 1.0 × 10^−8^–1.0 × 10^−2^ M with a limit of detection of 7.0 × 10^−9^ M. Whereas a microemulsion polymerized sensor had a Nernstian response (31.97 ± 0.6 mV decade^−1^) in a concentration range of 1.0 × 10^−7^–1.0 × 10^−2^ M and a detection limit of 8.0 × 10^−8^ M. Both electrodes showed high performance and long-term stability with a response time of 5 s [35].

For clinical analysis, Streptomycin recognition with an electrochemical sensor was achieved by composite of mercaptoacetic acid-modified PbS nanoparticles with Au-coated Fe_3_O_4_ nanoparticles dispersed in MWCNT-doped chitosan film. Imprinting of the template on a gold electrode was achieved by sol-gel polymerization and stepwise modification of nanocomposites. The sensor exhibited high selectivity and good electrocatalytic activity with a wide-range linear response in the range of 1.0 × 10^−6^–1.0 × 10^−3^ mol L^−1^ and a detection limit of 1.5 × 10^−9^ mol L^−1^ [66].

#### 3.1.2. Environmental Analysis

Huang *et al*. [60] reported a sensor for bisphenol A using MIP and Au-NPs. The strategy was successfully implemented by electropolymerization of 2-aminothiophenol on a Au-NP-modified glassy carbon electrode in the presence of a template. The designed sensor worked efficiently for a broad linear range, *i.e.*, 8.0 × 10^−6^–6.0 × 10^−2^ mol L^−1^, and demonstrated a low detection limit of 1.38 × 10^−7^ mol L^−1^ [60]. In an alternative approach by Huang *et al*. [61], a bisphenol A sensor was developed by fabricating thin films of molecularly imprinted sol-gel polymers. Composites of Au-NPs and multi-walled carbon nanotubes (MWCNTs) were used for efficient sensing of the analyte. A linear response was observed in the range of 1.13 × 10^−9^ mol L^−1^–8.21 × 10^−3^ mol L^−1^ with a detection limit of 3.6 × 10^−9^ mol L^−1^ [61]. Electrochemical recognition of trinitrotoluene (TNT) was achieved by Au-NPs composite film with conducting polymers that exhibited a detection limit as low as 46 ppt; thus, providing ultrasensitive detection of TNT [62]. Sensitive and selective cavities for dimethoate were developed on gold electrode by electropolymerization of o-phenylenediamine. Subsequently, Ag nanoparticles were deposited on the preformed film, which resulted in a layer height of 25 nm. The sensor showed an amperometric response towards the target analyte with a detection limit of 0.5 ng mL^−1^ [67].

#### 3.1.3. Food Quality/Process Analytics

Imprinted nanomaterials have also been successfully applied in food quality and process control industries. Zhao *et al*. [68] used imprinted core-shell nanoparticles with a silica core for the determination of tert-Butylhydroquinone in food stuffs. The silica core was modified with 3-chloropropyltrimethoxysilane and polyethylenimine and, subsequently, polymerized with EGDMA (ethylene glycol dimethacrylate). The resultant matrix demonstrated a high surface area, specificity and a linear response in the range of 0.1–50.0 mg/kg with a detection limit down to 0.27 mg/kg [68]. A sensor for erythrosine detection was fabricated on a gold electrode with chitosan-platinum nanoparticles and graphene-Au-NP nanocomposites. MIP film was formed by electropolymerization of HAuCl4, erythrosine and 2-mercaptonicotinic acid. The sensor showed a linear response in the concentration range of 7.0 × 10^−8^–9.0 × 10^−5^ mol L^−1^ and a low limit of detection, *i.e.*, 2.3 × 10^−8^ mol L^−1^. The sensor also exhibited good stability and sensitivity for detecting target species in real samples [69].

### 3.2. Optical Sensors

Optical sensors based on the fluorescent “turn-on” or “turn-off” behavior have been applied extensively for a variety of analytes. Recently, due to a more feasible measurement and signal output, many efforts have been made to develop optical sensors with high selectivity by using the molecular imprinting technique, which demonstrated encouraging results. Mostly, fluorescent ligands and fluorotag-ligands conjugates have been employed in the fabrication of these sensors, which display fluorescence enhancement or reduction upon analyte binding [6].

#### 3.2.1. Bioanalysis

Cubic MIP nanoparticles having a fluorescent-core yield highly selective receptor material, as they offer precise control over the number of fluorescent labels on each nanoparticle. Imp-NPs produced with these specifications are suitable for replacing natural antibodies in biosensors [80]. Imprinted nanoparticles containing the amino acid derivative, dansyl-L-phenylalanine, were entrapped in polymer nanofibers for their fluorescence-based biosensor application. Characterization of nanofibers for analyte binding was made by atomic force microscopy (AFM), optical microscopy and fluorescence microscopy, which showed high selectivity and good recovery [55]. A novel thiol ligand exchanged technique was used to recognize dipicolinic acid, which is a major constituent of *Bacillus cereus*. The sensor used methacryloyl amindoantipyrine-terbium as the metal chelating agent for manufacturing an imprinted nanostructure. The interaction between the metal ion and the free coordination sphere affected the binding ability of nanoclusters and nanosensors, respectively. Sensor responses were recorded as a function of decreasing fluorescence intensity [53].

In another study for dipicolinic acid recognition, methacryloyl iminodiacetic acid-chrome was used as the metal-chelating monomer in a novel thiol ligand-capping method having polymerizable methacryloylamidocysteine linked to Au-NPs. The interaction of the target analyte and the MIP nanosensor was observed by fluorescence measurements. The addition of the target analyte with MIP significantly decreased its fluorescence intensity, due to the induction of photoluminescence emission from Au-NPs. The change in fluorescence intensity was attributed to strong complexation with the analyte [54]. In another approach, polymerizable methacryloylamidocysteine linked to Au-Ag nanoclusters with a reconstructed surface shell by molecular imprinting were used. Methacryloyl iminodiacetic acid-chrome was used as the metal-chelating monomer and dipicolinic acid as the target analyte. The sensor exhibited decreased fluorescence intensity, due to a high complexation of the analyte on the surface of Au-Ag nanoshells, and good affinity with the target specie [71].

Imprinted nanospheres have been applied for label-free direct optical sensing of small molecules. Imprinted L-boc-phenylalanine anilide nanospheres were generated via miniemulsion polymerization and immobilized on an aminosilane layer. The sensor showed a rapid response, good selectivity, high reversibility, with a detection limit down to 60 μM, and a limit of quantitation of 94 μM. The MIP-based sensor exhibited significant long-term stability, since no loss in signal intensity was observed after storing them for one year [51].

**Figure 5:**
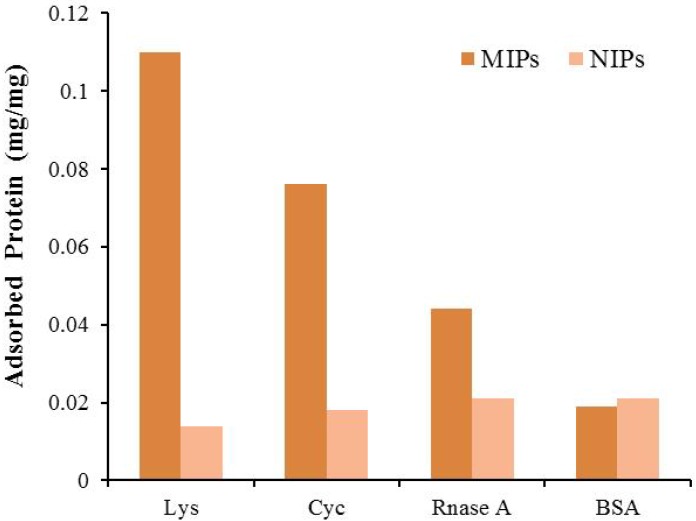
Comparison of different imprinted and non-imprinted polymers (NIP) to evaluate the adsorption selectivity for lysozyme (Lys), cytochrome C (Cyc), ribonuclease A (RNase A) and bovine serum albumin (BSA), respectively, at a concentration of 0.5 mg mL^−1^ for all. Adopted from [73].

Different researchers developed optical sensors using Imp-NPs for the recognition of biomolecules [72,73]. In one approach, fluorescence changes were observed in embedded quantum dots to analyze salivary secretion molecules, *i.e.*, amylase, lipase and lysozyme. A quantum dot composite with an imprinted polymer was synthesized by phase inversion of poly(ethylene-co-vinyl alcohol) solutions with different ethylene molar ratios. Quantum dots exhibited fluorescence quenching upon exposure to analytes, and this response was used for the quantitative recognition of amylase, lipase and lysozyme in saliva [72]. Similarly imprinted multifunctional lysozyme Imp-NPs were synthesized [73] by encapsulation of Fe_3_O_4_ nanoparticles. These magnetic nanoparticles possessed high adsorption capacity towards the target analyte, as shown in Figure 5.

The controlled selectivity and the ease of separation from the crude sample made it a sensitive chemiluminescence scheme for the recognition of lysozyme in human serum. Imp-NPs can be synthesized by a novel thiol ligand-capping technique with polymerizable methacryloylamido-cysteine. DNA recognition was achieved successfully by using nanoparticles linked to a CdS quantum dots reconstructed surface shell by synthetic host polymerization. Methacryloylamidohistidine-platinium was applied as a metal-chelating monomer. These nano-shell sensors responded efficiently to guanosine compared to its analogous molecules, as observed through fluorescence intensity enhancement [52].

#### 3.2.2. Environmental Analysis

Selective recognition of naphthalene in vapor form has been observed by a layer of core-shell polymer nanoparticles obtained via imprinting technology. A pseudo-second order kinetic approach for fluorescent analysis was employed, which showed selective recognition of the target analyte with a high rate constant [74].

The electrochemiluminescence method for selective recognition of thifensulfuron-methyl (TFM) using core-shell Imp-NPs was successfully employed. Chitosan composite film with surface imprinted silica nanoparticles was deposited on the surface of a glassy carbon electrode. After eradicating silica cores from the composite film, the sensor response was investigated in the presence and absence of the analyte. Electrochemiluminescence intensity enhanced remarkably by 2.7-folds in the presence of the target species. The sensor exhibited a linear response in the range of 5.0 × 10^−10^–1.0 × 10^−7^ M with a low detection limit of 0.32 nM. The proposed method provided a promising scheme for sulfonylurea herbicide recognition [70].

#### 3.2.3. Food Quality/Process Analytics

A sensing system based on Imp-NPs for direct analysis of fluoroquinolone in food and environmental samples was developed using enrofloxacin (ENRO) as a model template. Imp-NPs were successfully applied for the first time to analyze fluoroquinolone in real samples. The sensor exhibited a lower detection limit down to 0.1 nM for enrofloxacin with 5 μg mL^−1^ of MIP. In milk, enrofloxacin and danofloxacin, whose maximum residue limits have been set to 0.28 μM and 0.08 μM, respectively, could be detected with high selectivity. This method provides rapid and robust on-site analysis based on fluorescence polarization measurements [81].

A flow injection chemiluminescence sensor was designed for the recognition of chrysoidine using magnetic MIPs as the recognition element, *i.e.*, developing a Fe_3_O_4_ layer on SiO_2_ particles. Synthesized nanoparticles showed specific recognition with enhanced adsorption capacity and easy magnetic separation. The linear response was observed for the target analyte in the range of 1.0 × 10^−4^–2.0 × 10^−6^ mol L^−1^ with a detection limit of 6.19 × 10^−7^ mol L^−1^. The sensor was highly sensitive and reusable for chrysoidine determination in food samples [39].

### 3.3. Mass Sensitive Devices

Acoustic or mass-sensitive devices are used to measures the mass of a target analyte, which is a universal property of every substance. Quartz crystal microbalance (QCM) is a highly sensitive mass measuring piezoelectric device that works by decreasing the oscillation frequency of a piezoelectric crystal upon mass loading. According to the Sauerbrey equation, the change in frequency (sensor response) is directly proportional to the square of the fundamental resonance frequency. Piezoelectric devices are universally applicable with low cost, a good detection limit and the ease of assembling [6]. We provide here some exemplary applications of imprinted nanomaterial for mass sensitive devices.

#### 3.3.1. Bioanalysis

A QCM sensor for 17β-estradiol was fabricated by depositing Imp-NPs obtained via miniemulsion polymerization on a gold electrode of a QCM sensor. The specificity of designed sensor was examined through comparative adsorption of 17β-estradiol, stigmasterol and cholesterol, which exhibited higher selectivity towards the target analyte. The sensor exhibited a linear response in a broad range of 3.67 nM–3.67 pM. The quantification and limit of detection were 2.04 pM and 613 fM, respectively. The proposed imprinted QCM sensor [75] had a good long-lasting storage stability, resistance to microbial growth, robustness and reusability and, therefore, was cost effective. In a related study, mass-sensitive detection of folic acid was performed with the help of molecularly imprinted films and nanoparticles. Methacrylate and acrylate-vinyl pyrrolidone copolymers were used for binding the analyte. Imp-NPs showed a rapid response and high affinity as compared to the MIP film obtained from methacrylate polymers, as the sensitivity was enhanced by a factor of three [76].

Lysozyme Imp-NPs were synthesized by miniemulsion polymerization for the fabrication of a QCM nanosensor. Nanoparticles were fixed on a gold surface by coating and then dried at 37 °C for about six hours. The Imp-NP monolayer film showed an appreciably low detection limit of 1.2 ng mL^−1^. Albumin was used to study specificity. The QCM nanosensor demonstrated outstanding sensitivity and selectivity for the target analyte in both natural sources, *i.e.*, egg white (460–1500 ng/mL) and aqueous solutions (0.2–1500 μg/mL) [77]. Another interesting application of these nanomaterials is developing affinity-based Imp-NPs. Zeng *et al*. [78] used this strategy for peptide sensing by incorporating a target protein in a polymer matrix. The peptide analyte was coupled with fatty acids of varying chain length. The resulting nanoparticles showed binding affinity in the range of 90–900 nM, when evaluated by QCM [78].

#### 3.3.2. Environmental Analysis

**Figure 6:**
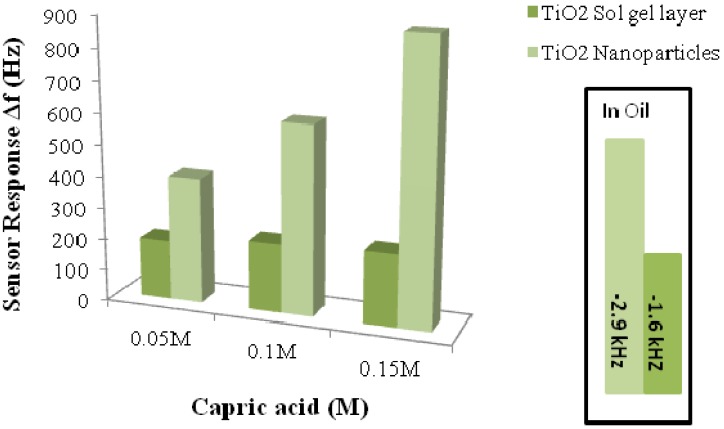
Comparison of the QCM sensor responses for the imprinted titania layer and nanoparticles to different capric acid concentrations. The inside graph shows the frequency responses when shifted from fresh oil to waste oil; nanoparticle electrode offers a better response. Adopted from [82].

A mass sensitive sensor based on imprinted TiO_2_ nanoparticles deposited on QCM for engine oil degradation sensing was fabricated by Lieberzeit *et al*. [42]. The size of imprinted titania particles was in the range of 200–300 nm. Imp-TiO_2_-NPs showed enhanced uptake of capric acid, *i.e.*, the template, in comparison with the corresponding sol-gel films, thus making this approach highly compatible for industrial processes [42]. The same group later explored further strategies based on imprinted titania materials, *i.e.*, TiO_2_ nanoparticles or sol-gel layers, for comparative analysis and monitoring of oxidized compounds in lubricants [82,83,84]. Imp-TiO_2_-NPs responded twice as high as titanate thin films, because of the higher surface area and easier diffusion within the imprinted sites of nanoparticles as compared to thin films. Figure 6 clearly shows the response of nanoparticles, which is almost double compared to that of the titanate sol-gel layer at different concentrations of capric acid.

Lieberzeit and co-workers [82,85], in their recent studies, revealed that soft nanomaterials, like MoS_2_, bear great affinity towards thiols. They presented the expedited sensor signals, *i.e.*, more than 10-times higher than bare gold electrodes [82], achieved, and a detection limit beyond 0.5 ppm [85] was achieved.

Imprinted nanocomposite materials are new entrants in this field, especially when combined with mass-sensitive devices; these materials can have a number of applications. Iqbal *et al*. [86], in their recent experimentation, used imprinted polyurethane (PU) Au-NPs composite material for the selective detection of some common volatile organic compounds (used as lab reagents) down to a few ppm. The interesting feature of their strategy was the synergistic effect of molecular imprinting and nanoscale gold on sensor response and selectivity, which was achieved by *in situ* mixing of Au-NPs in the imprinted PU material.

#### 3.3.3. Food Quality/Process Analytics

A number of sensing devices have been reported for monitoring food quality and for process control [87,88]. Mass-sensitive devices stand alone among them, due to their application to almost any kind of analyte. MIPs in joint venture with mass sensitive devices have been reported [89,90] for chemical analysis of food and processes. In a recent study, Kong *et al*. [91] fabricated a QCM sensor imprinted with polymers and Au-NPs for the detection of Ractopamine (RAC). RAC is a common β-adrenergic agonist that is widely used in animal feed as a growth promoting agent, because it can increase the percentage of lean meat and improve feed conversion ratios. Yet, its presence in animal tissues and meat products is potentially hazardous for living beings. Hence, its monitoring is vital. Kong *et al*. electrodeposited molecularly imprinted a poly(o-aminothiophenol) (PoAT) membrane on an Au electrode surface modified by self-assembled Au-NPs. The analytical measurements revealed that integration of Au-NPs into MIP via an electro-polymerization process could improve the performance, *i.e.*, the sensitivity and selectivity, of the imprinted electrodeposited membrane [91].

## 4. Outlook and Future Perspectives

Molecularly imprinted nanomaterials have shown tremendous potential for the recognition of a wide variety of analytes, ranging from small molecules to large proteins and macromolecules. Combining imprinting with nanotechnology appreciably boosted up both sensitivity and selectivity. The large surface area offered by imprinted nanostructures exposed more binding sites to attract the target analyte. Molecularly imprinted nanomaterials of varying sizes and dimensions can be synthesized through various methodologies, *i.e.*, precipitation, emulsion, bulk, the core-shell approach, electrospinning, *etc.* This review focused on applications of imprinted nanomaterials with an emphasis on electrochemical, optical and mass-sensitive detection of the desired analytes. These nanomaterials were further categorized into subgroups, like bioanalytes, environmental and food/industry application, with details and examples, so that the reader may develop an understanding of the topic. The nanosized dimensions of the sensing interface provide a large surface area that can comprehend plenty of interactions sites between the sensitive layer and the analyte, and thus, amplifies the sensor signal. Secondly, it triggers better selectivity, due to the adopted cavities, with ease of access to binding sites for targets, which offer the rapid response and recognition of target species. Nanosized MIPs have well-defined dimensions, and their exposed imprinted sites provide rapid analyte transfer. Furthermore, the selectivity of molecular imprinted nanoparticles exhibited in electrochemical and optical sensors can be enhanced by improving the binding of the layer with the transducer surface and developing a straightforward understanding of the primary signal transduction mechanism.. Molecular imprinted nanomaterials are more potable in sensing devices, which could help in addressing on-field chemical analysis and remote sensing, as well. For better performance, there are certain problems that need to be resolved, e.g., the non-uniformity of binding sites, the lack of a general synthesizing protocol and improper adherence with the transducer surface. In order to make such chemical sensors commercially viable, the MIP layer and transducer properties need to be tuned. Exploring composite and hybrid organic inorganic polymers for developing imprinted materials in chemical sensing, e.g., for the food and processing industry, would be of great value.
